# The Second Look: The Future of Surgical Education

**DOI:** 10.7759/cureus.109608

**Published:** 2026-05-25

**Authors:** Mohammad Safadi, Wisam Abboud, Amir Farah

**Affiliations:** 1 General Surgery, Nazareth Hospital EMMS, Nazareth, ISR; 2 Surgery, Medical College of Wisconsin, Milwaukee, USA

**Keywords:** general surgery, laparoscopic surgery, procedural education, residency program, simulation in medical education

## Abstract

Operative video review is increasingly being recognized as a valuable tool in surgical education. By providing an objective and reproducible record of intraoperative performance, video review allows trainees and educators to revisit technical maneuvers, decision-making, communication, and operative flow in ways not possible through memory alone. Beyond identifying errors, video analysis can help reveal the subtle cognitive and technical behaviors that characterize surgical expertise.

Video-based assessment also offers the potential for more objective evaluation of surgical competency and deliberate practice. As digital technologies and artificial intelligence continue to evolve, operative video review may become an increasingly important component of modern surgical training. Its successful implementation, however, will require careful attention to confidentiality, psychological safety, and educational culture.

## Editorial

Video review has transformed nearly every performance-based profession. Athletes study game film. Pilots analyze cockpit recordings. Military teams conduct structured after-action reviews. Yet surgery, despite being one of the most technically and cognitively demanding disciplines in medicine, has historically relied on memory, subjective feedback, and retrospective recollection to evaluate operative performance. This paradigm is beginning to change.

Surgical education has long followed the apprenticeship model of “see one, do one, teach one" [1]. While this framework has produced generations of excellent surgeons, it is increasingly challenged by modern realities: work-hour restrictions, growing procedural complexity, variability in operative exposure, and heightened expectations for patient safety and measurable competency. In this environment, video review offers an opportunity not merely to supplement surgical training, but to fundamentally redefine it [2].

The operating room is a uniquely information-dense environment. Technical maneuvers, communication patterns, leadership decisions, situational awareness, instrument handling, team coordination, and responses to intraoperative adversity unfold simultaneously and often rapidly. Much of this complexity is lost once the operation ends. Operative notes capture outcomes and key procedural steps, but they cannot fully reconstruct the nuances of performance. Human memory is selective and imperfect, especially after stressful or prolonged operations. Video review creates an objective record that allows surgeons to revisit these moments with clarity [3].

Importantly, video review is not solely about identifying errors. One of its greatest educational strengths lies in revealing the invisible components of expertise. Experienced surgeons often perform critical cognitive and technical tasks automatically, which provides a steep learning curve to master moments that reflect expert performance from mere task completion. Reviewing operative footage allows educators to slow down decision-making, examine economy of motion, analyze operative flow, and discuss alternative strategies in a way that is rarely achievable in real time during surgery.

This is of particular importance in the non-elective setting, where chaos ensues, and memory of the acute nature of the procedure and clinical care may not be fully captured. Video review also introduces the possibility of a more objective assessment in surgical training. Traditional operative evaluations remain vulnerable to inconsistency, recall bias, and interpersonal dynamics. Two trainees performing similarly may receive vastly different assessments depending on faculty expectations or case circumstances. Structured video-based assessment may improve fairness and reproducibility by allowing multiple reviewers to evaluate the same performance using standardized criteria. Beyond technical skill, video analysis may help quantify domains such as leadership, communication, operative sequencing, and efficiency.

At the same time, the expansion of video review raises legitimate concerns. Surgeons may fear punitive use, medicolegal exposure, or erosion of trust within training environments. These concerns cannot be dismissed. Programs implementing video review must establish clear safeguards regarding confidentiality, consent, data ownership, and educational intent. The goal should not be surveillance. It should be growth. A culture of psychological safety is essential if surgeons and trainees are to engage honestly with performance review.

Another challenge is avoiding reductionism. Not every aspect of surgical judgment can be captured by metrics or timestamps. Surgery remains deeply human, requiring intuition, adaptability, and contextual decision-making. Video review should augment mentorship rather than replace it. The most valuable lessons often emerge not from the footage itself, but from the discussion surrounding it between trainees and experienced surgeons.

Artificial intelligence (AI) may further expand the role of operative video analysis [3]. Emerging technologies may eventually identify operative phases automatically, measure instrument motion, evaluate efficiency, detect deviations from standard workflow, or provide individualized feedback. Such systems hold promise, but caution is warranted. Surgical performance is highly context dependent, and simplistic metrics risk misunderstanding complexity. AI should assist educators, not replace expert judgment.

The future of surgical education will likely be increasingly data-driven, reflective, and multimedia-based. Video review represents one of the clearest opportunities to bridge the gap between operative experience and deliberate practice. Surgery has traditionally depended on what surgeons remember. Video review allows the field to learn from what actually happened.

The operating room has always been a classroom. Video review may finally allow us to study it with the rigor it deserves.

## References

[1] Ayub SM (2022). "See one, do one, teach one": balancing patient care and surgical training in an emergency trauma department. J Glob Health.

[2] Balvardi S, Kammili A, Hanson M (2022). The association between video-based assessment of intraoperative technical performance and patient outcomes: a systematic review. Surg Endosc.

[3] Dick L, Boyle CP, Skipworth RJE, Smink DS, Tallentire VR, Yule S (2024). Automated analysis of operative video in surgical training: scoping review. BJS Open.

